# Admission Hyperglycemia Predicts Long-Term Mortality in Critically Ill Patients With Subarachnoid Hemorrhage: A Retrospective Analysis of the MIMIC-III Database

**DOI:** 10.3389/fneur.2021.678998

**Published:** 2021-10-05

**Authors:** Dongliang Liu, Yiyang Tang, Qian Zhang

**Affiliations:** ^1^Department of Spine Surgery, Xiangya Hospital, Central South University, Changsha, China; ^2^Department of Cardiology, Xiangya Hospital, Central South University, Changsha, China; ^3^Department of Neurosurgery, Xiangya Hospital, Central South University, Changsha, China

**Keywords:** subarachnoid hemorrhage, hyperglycemia, mortality, MIMIC III database, prognosis

## Abstract

Subarachnoid hemorrhage (SAH) is a severe subtype of stroke with high mortality. Hyperglycemia is a common phenomenon in critically ill patients and associated with poor clinical outcome. However, the predictive value of admission hyperglycemia for 30 and 90-day all-cause mortality in critically ill patients with SAH remains controversial. All SAH patients between 2001 and 2012 were included based on the MIMIC-III database and were further classified according to the tertiles of blood glucose (BG) measured on intensive care unit (ICU) admission. Clinical information including demographic data, comorbidities, and laboratory indicators were exacted and analyzed. The primary outcomes were 30- and 90-day all-cause mortality. A total of 1,298 SAH patients were included. The 30 and 90-day mortality rates were 19.80% and 22.73%, respectively. Subjects in the high glucose tertile were older, were overweight, had higher sequential organ failure assessment (SOFA) and Simplified Acute Physiology Score II (SAPS II) scores, and presented higher mortality rate. Generalized additive model revealed a U-shaped relationship between BG and 30 and 90-day all-cause mortality. Furthermore, Kaplan–Meier (K-M) survival curve also illustrated that subjects with admission hyperglycemia presented lower survival rate and shorter survival time. In Cox analysis, after adjustment for potential confounders, admission hyperglycemia was related to an increase in 30- and 90-day all-cause mortality in SAH patients. In subgroup analysis, the association between admission hyperglycemia and all-cause mortality was consistent. In conclusion, admission hyperglycemia is associated with significantly increased 30- and 90-day all-cause mortality in critically ill patients with SAH.

## Introduction

Subarachnoid hemorrhage (SAH), mainly caused by intracranial aneurysm, is a severe subtype of stroke that carries high mortality (1, 2). The estimated incidence of SAH is 6–20 per 100,000 (3). SAH is fatal and usually strikes at a fairly young age, which makes the loss of productive life years of SAH as serious as that of intracerebral hemorrhage or cerebral infarction, although only one in every 20 strokes is caused by SAH (1). The fatality rate is 8.3–66.7% in patients with SAH, and ~8.3% of them die before getting to the hospital; 40% of hospitalized patients die within 1 month after the event; and more than 30% of those survivors suffer from major neurologic deficits (4). The clinical outcome of SAH depends on the severity of the initial hemorrhage; but other factors at hospital admission such as older age, history of hypertension, larger aneurysm, and posterior circulation aneurysm also play an important role in the clinical prognosis (5). However, only 25% of the variation outcome could be explained by these reported variables, indicating that other unknown factors might have substantial effects on the clinical outcome (5, 6). Exploring predictors of the short- or long-term prognoses, especially if these could be modified, has great clinical significance because this would provide the potential to improve treatment and prognosis.

As one of the most commonly used biomarkers, blood glucose (BG) provides important clues regarding the diagnosis or prognosis of various disorders, including traumatic shock (7), hypertension (8), infection after total joint arthroplasty (9), and liver transplantation (10). Hyperglycemia was independently associated with delayed cerebral ischemia (DCI) and poor outcome in SAH (11–14); contrarily, lower admission BG level was related to neurological grade improvement (15). However, a later study reported that lactate and glucose were strongly related (Spearman ρ = 0.55; *p* < 0.001), BG was only independently associated with DCI, while lactate was independently associated with poor outcome with multivariable analyses in a prospective study of 285 SAH patients (16).

The relationship between admission hyperglycemia and mortality was also extensively analyzed; Bian et al. demonstrated that admission BG was associated with 1-year mortality after being adjusted for other confounding factors in a study including 239 SAH patients (17). Another study from South Korea including 553 SAH patients found that BG at admission was not an independent predictor for 3-month mortality, although BG level was significantly higher in non-survivors and patients with poor outcome (18). Incidentally, there were also studies suggesting that admission hyperglycemia predicted short-term (30 days), but not long-term, mortality in SAH patients (19). Due to the controversy of current evidence and limited sample size, admission BG has not been widely considered as an independent risk factor for poor outcome after aneurysmal SAH (3, 6, 20–22). Based on Medical Information Mart for Intensive Care (MIMIC) III database (12, 13), the objective of the present study is to determine the effect of admission hyperglycemia on the prognosis in critically ill SAH patients and also to identify a threshold for admission BG levels that predicts unfavorable outcome in SAH.

## Materials and Methods

### Data Source

This is a retrospective study based on an openly available MIMIC III (version 1.4) database (12, 13), which is a large, single-center database compiling the clinical data of 46,520 critically ill patients admitted to intensive care unit (ICU) at the Beth Israel Deaconess Medical Center (Boston, Massachusetts) between 2001 and 2012 (23). In this study, all ICU admissions with SAH were included based on MIMIC III database. To access the database, the National Institutes of Health's web-based course “Protecting Human Research Participants” (No. 9014457) was completed.

This study was approved by the Institutional Review Boards of Beth Israel Deaconess Medical Center and the Massachusetts Institute of Technology (Cambridge, MA). To protect patient privacy, all data were de-identified; thus, informed consent was waived by the ethical committee of the Beth Israel Deaconess Medical Center.

### Selection of Participant

**Figure 1:**
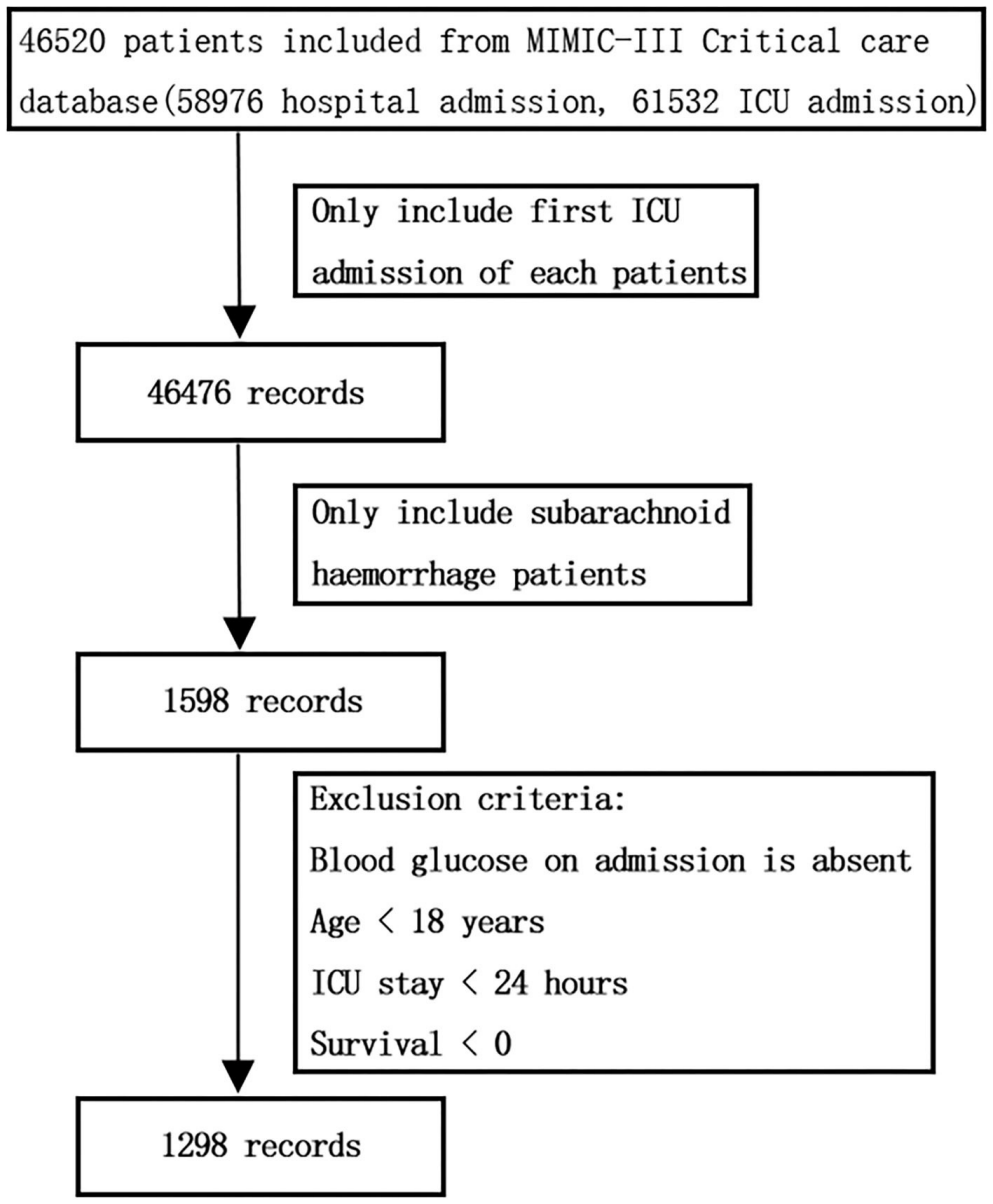
Flow chart of data extraction. ICU, intensive care unit.

Based on the ninth revision of the International Classification of Diseases code, SAH patients with age ≥18 years in the MIMIC-III database were included for analysis. For patients who were admitted to the ICU multiple times, only the first ICU admission data were included. Patients without any BG data within 24 h after admission or with individual data missing rate >5% were not included. ICU patients with length of stay <24 h were also excluded to avoid potential extremum value influence. The workflow is shown in Figure 1.

### Data Extraction

Structured Query Language (SQL) with PostgreSQL (version 9.6) was used to extract baseline characteristics, vital signs, comorbidities, laboratory variables, and others within the first 24 h after ICU admission from MIMIC-III database. Baseline characteristics included age, gender, ethnicity, and weight. Severity at admission was measured by the sequential organ failure assessment (SOFA) score, the Simplified Acute Physiology Score II (SAPS II), and Glasgow Coma Scale (GCS). The use of mechanical ventilation, application of dialysis, and administration of vasopressors were also recorded. Vital signs included the systolic blood pressure (SBP), diastolic blood pressure (DBP), mean blood pressure, heart rate, temperature, respiratory rate, and percutaneous oxygen saturation (SpO_2_). Comorbidities including liver disease, renal failure, cardiac arrest, congestive heart failure, hypertension, diabetes mellitus (DM), pneumonia, respiratory failure, and malignancy were also collected for analysis based on the recorded International Classification of Diseases code-9 from the MIMIC-III database. Laboratory variables were collected within the first 24 h after admission including white blood cell count, hemoglobin, platelet counts, BG, creatinine, blood urea nitrogen, anion gap, sodium, potassium, chloride, bicarbonate, prothrombin time, and activated partial thromboplastin time.

### Grouping and Outcomes

To explore the effect of glucose on the prognosis of critically ill patients with SAH, all enrolled subjects were stratified according to the tertiles of admission glucose values, namely, the low glucose group (tertile 1, BG ≤ 122.00 mg/dl or BG ≤ 6.80 mmol/L), the middle glucose group (tertile 2, 122.00 < BG <155.33 mg/dl or 6.80 mmol/L < BG <8.65 mmol/L), and the high glucose group (BG ≥ 155.33 mg/dl or BG ≥ 8.65 mmol/L). Follow-up of patients began from the day of admission and ended at death. The primary outcomes of interest were 30-day, 90-day, and in-hospital all-cause mortality.

### Statistical Analysis

All statistical analyses were performed on EmpowerStats version 2.20 (http://www.empowerstats.com/cn/, X&Y Solutions, Inc., Boston, MA, USA) and R software version 3.4.3. Continuous variables were presented as mean ± standard deviation (SD) or median ± interquartile range (IQR) and analyzed by the Kruskal–Wallis test; categorical variables were presented as number and percentage and analyzed by chi-square (or Fisher's exact) tests. Multiple imputations with multivariate imputation by chained equation were used for handling missing values. Variables with missing rate over 20% were converted to dummy variables in the models to avoid possible bias caused by direct filling missing values.

Generalized additive model (GAM) is a non-parametric statistical model that directly deals with the non-linear relationship between multiple explanatory variables and response variables (24, 25). GAM was used in this study to determine the non-linear association between BG and 30- and 90-day all-cause mortality in critically ill patients with SAH. The Kaplan–Meier (K-M) curve followed by log-rank test was utilized to visualize the relationship. Furthermore, the Cox proportional hazards model was performed to determine the association between BG and 30- and 90-day all-cause mortality in these patients, using the first tertile as the reference. Two multivariate models were conducted to adjust potential confounders in the Cox proportional hazards model, and results were presented as hazard ratios (HRs) and 95% confidence intervals (CIs). Baseline variables that were considered clinically relevant or change in effect estimate exceeding 10% (26) were chosen as confounders, including age, sex, and ethnicity in model I. Based on model I, we further included heart rate, cardiac arrest, SBP, DBP, SOFA, SAPS II, GCS, anion gap, white blood cell, bicarbonate, dialysis, and mechanical ventilation as confounders in our model II.

Subgroup analysis on the correlation between BG and 30-day all-cause mortality was further conducted based on their comorbidities (liver disease, hypertension, DM, pneumonia, respiratory failure, and congestive heart failure), treatment (vasopressor and ventilation utilization), and disease severity scores (SOFA, SAPS II, and GCS) in critically ill patients with SAH. A two-sided *p* < 0.05 was considered statistically significant.

## Results

### Baseline Characteristics of Subjects

MIMIC-III database comprises 46,520 patients admitted to the ICU of Beth Israel Deaconess Medical Center; among them, 1,590 patients were diagnosed with SAH. After exclusion of patients who were younger than 18 years old, ICU stay <24 h, missing glycemic data, and survival time <0, a total of 1,298 patients (601 male and 697 female) were included in the present study with an average age of 57.64 ± 19.79 (18–89 years old). The detailed information of patient selection is presented in Figure 1.

The 30- and 90-day mortality rates for the overall subjects were 19.80% (257) and 22.73% (295), respectively. The baseline characteristics of enrolled subjects stratified by admission BG tertiles are summarized in Table 1. There were 419 subjects in the low glucose group, and 451 and 428 SAH patients in the middle glucose group and high glucose group, respectively. Overall, subjects with hyperglycemia were older; were overweight; had more comorbidities of DM, pneumonia, renal failure, and respiratory failure; and had higher SOFA and SAPS II scores. They presented higher 30- and 90-day mortality rates, were more likely to have abnormal laboratory test (such as increased white blood cell, platelet, and creatinine), and have higher rates of vasopressor and mechanical ventilation utilization.

**Table 1:** The clinical characteristics of critically ill patients with subarachnoid hemorrhage based on admission blood glucose tertile.

**Characteristics**	**Glucose (mg/dl)**			***P*-value**
	**Tertile 1 (*n* = 419)**	**Tertile 2 (*n* = 451)**	**Tertile 3 (*n* = 428)**	
Age (years)	53.27 ± 20.91	57.32 ± 19.42	62.25 ± 17.99	<0.001
Gender, *n* (%)				0.347
Male	182 (43.44)	217 (48.12)	202 (47.2)	
Female	237 (56.56)	234 (51.88)	226 (52.8)	
Ethnicity, *n* (%)				0.056
White	308 (73.51)	326 (72.28)	284 (66.36)	
Black	21 (5.01)	26 (5.76)	19 (4.44)	
Other	90 (21.48)	99 (21.95)	125 (29.21)	
Weight, kg	75.04 ± 18.73	76.97 ± 18.52	78.93 ± 21.11	0.026
SBP, mmHg	124.02 ± 13.61	126.11 ± 12.83	125.05 ± 13.61	0.067
DBP, mmHg	63.34 ± 9.38	63.36 ± 9.78	61.24 ± 9.38	0.001
MBP, mmHg	81.21 ± 9.41	82.58 ± 9.65	80.86 ± 9.18	0.030
HR, beats/min	79.53 ± 13.85	82.20 ± 15.28	83.68 ± 14.09	<0.001
RR, beats/min	17.49 ± 3.0	17.65 ± 3.23	18.62 ± 3.47	<0.001
Temperature, °C	37.1 ± 0.57	37.1 ± 0.58	37.06 ± 0.56	0.529
SpO_2_, %	97.79 ± 1.69	98.04 ± 1.71	97.99 ± 1.89	0.019
Length of stay	125.9 ± 152.3	186.7 ± 194.4	197.8 ± 195.0	<0.001
Endovascular coil occlusion, *n* (%)	2.6 (11)	4.9 (22)	5.6 (24)	0.078
Endovascular embolization, *n* (%)	3.1 (13)	2.2 (10)	2.3 (10)	0.701
**Scoring systems**				
SAPS II	27.48 ± 11.6	31.14 ± 12.12	36.12 ± 13.73	<0.001
SOFA	2.67 ± 2.14	3.06 ± 2.18	3.75 ± 2.68	<0.001
GCS	13.28 ± 2.78	12.98 ± 3.02	13.02 ± 3.28	0.206
Dialysis, *n* (%)	2 (0.5)	5 (1.1)	6 (1.4)	0.369
Vasopressor, *n* (%)	41 (9.6)	65 (14.7)	75 (17.4)	0.003
Ventilation, *n* (%)	168 (39.2)	215 (48.8)	269 (62.6)	<0.001
30-Day mortality, *n* (%)	42 (10.0)	72 (16.0)	143 (33.4)	<0.001
90-Day mortality, *n* (%)	54 (12.9)	82 (18.2)	159 (37.2)	<0.001
**Comorbidities**, ***n*** **(%)**				
Liver diseases	17 (4.0)	17 (3.9)	16 (3.7)	0.983
Renal failure	11 (2.6)	10 (2.3)	28 (6.5)	0.001
Cardiac arrest	3 (0.7)	11 (2.5)	12 (2.8)	0.060
Congestive heart failure	27 (6.3)	30 (6.8)	44 (10.2)	0.063
Hypertension	15 (3.5)	13 (3.0)	24 (5.6)	0.113
Diabetes	22 (5.1)	32 (7.3)	113 (26.3)	<0.001
Pneumonia	51 (11.9)	67 (15.2)	82 (19.1)	0.014
Respiratory failure	36 (8.4)	67 (15.2)	79 (18.4)	<0.001
Malignancy	6 (1.4)	6 (1.4)	9 (2.1)	0.630
**Laboratory tests**				
WBC (K/μl)	10.94 ± 5.01	12.45 ± 5,64	14.39 ± 6.33	<0.001
Platelet (K/μl)	210.58 ± 74.41	222.24 ± 81.90	230.18 ± 94.90	0.007
Hemoglobin (g/dl)	11.96 ± 1.81	11.83 ± 1.93	11.7 ± 2.13	0.132
Creatinine (mg/dl)	0.83 ± 0.33	0.84 ± 0.36	0.93 ± 0.44	<0.001
BUN (mg/dl)	13.56 ± 7.27	15.18 ± 8.15	17.7 ± 10.45	<0.001
Anion gap (mmol/L)	13.77 ± 2.79	14.07 ± 2.86	15.42 ± 3.51	<0.001
Sodium (mmol/L)	139.52 ± 4.02	139.58 ± 4.24	139.04 ± 4.3	<0.014
Potassium (mmol/L)	3.9 ± 0.56	3.93 ± 0.54	4.01 ± 0.65	0.016
Chloride (mmol/L)	105.77 ± 4.87	106.04 ± 5.31	105.20 ± 5.44	0.053
Bicarbonate (mmol/L)	23.99 ± 3.34	23.47 ± 3.41	22.48 ± 3.64	<0.001
PT (s)	13.30 ± 2.68	13.51 ± 2.86	14.16 ± 3.89	<0.001
APTT (s)	27.02 ± 7.66	27.36 ± 12.96	28.47 ± 15.35	0.027

*SAH, subarachnoid hemorrhage; SBP, systolic blood pressure; DBP, diastolic blood pressure; MBP, mean blood pressure; RR, respiratory rate; HR, heart rate; SpO_2_, percutaneous oxygen saturation; SOFA, sequential organ failure assessment; SAPS II, simplified acute physiology score II; GCS, Glasgow Coma Scale; WBC, white blood cell; BUN, blood urea nitrogen; PT, prothrombin time; APTT, activated partial thromboplastin time*.

### Association Between Admission Blood Glucose and All-Cause Mortality in Subarachnoid Hemorrhage Patients

**Figure 2:**
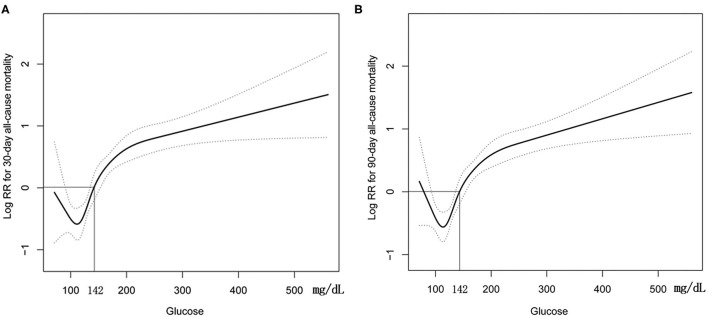
Construction of smooth curve describing the risk of mortality against admission BG using a generalized additive model. **(A)** 30-day all-cause mortality; **(B)** 90-day all-cause mortality. Dashed curves present the 95% confidence interval.

**Figure 3:**
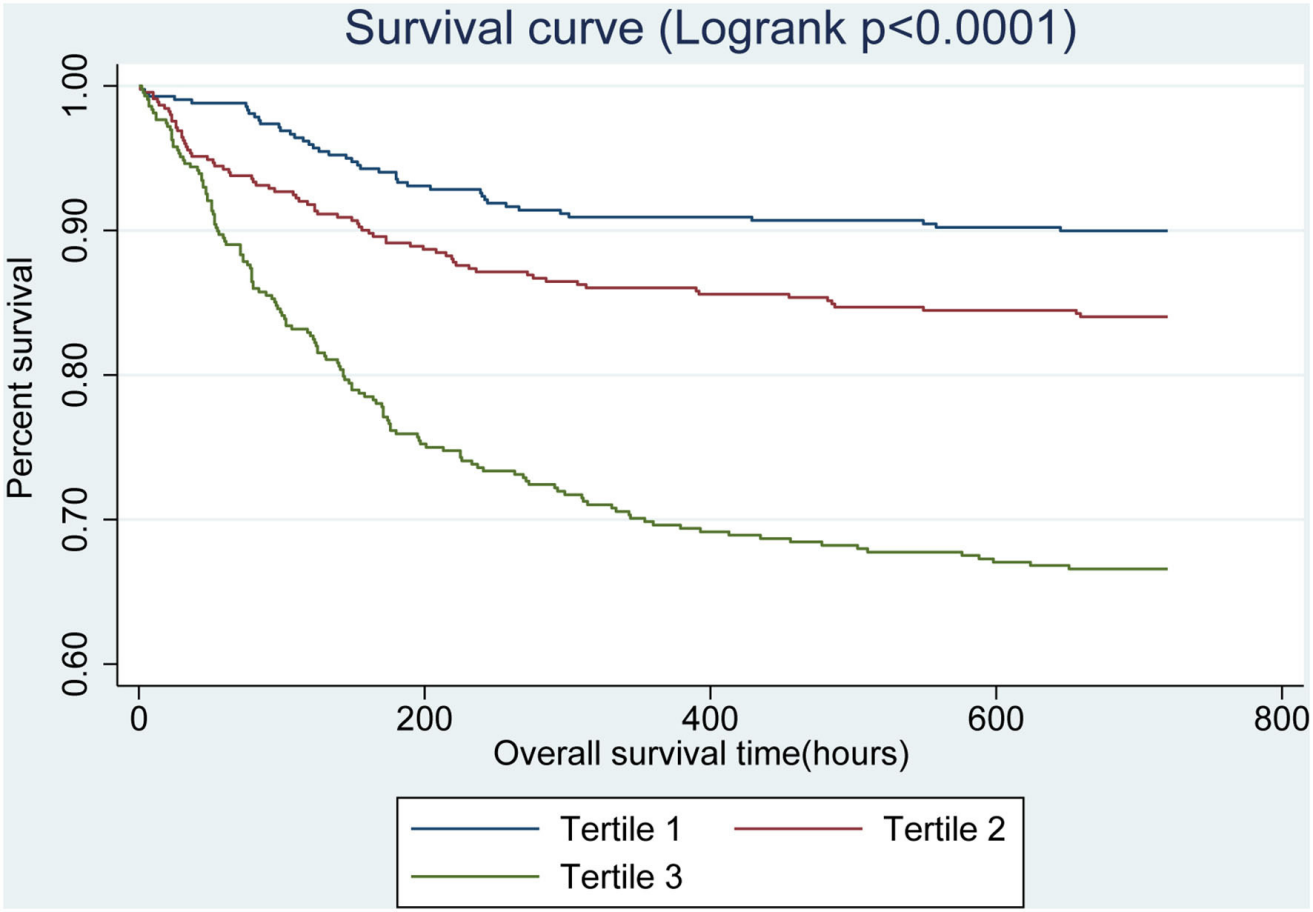
Kaplan–Meier survival curves for critically ill patients with SAH based on tertile of admission BG. x-Axis: survival time (h). y-Axis: cumulative survival probability. SAH, subarachnoid hemorrhage. BG, blood glucose.

GAM analysis revealed a U-shaped relationship between admission BG and 30-day all-cause mortality in patients with SAH, which is consistent in 90-day all-cause mortality. As shown in Figures 2A,B, subjects with BG <142.00 mg/dl (BG <7.91 mmol/L) were associated with a lower risk of 30- and 90-day all-cause mortality. With the further increase of BG (BG ≥142.00 mg/dl), there is an increase of the 30- and 90-day all-cause mortality in critically ill patients with SAH. Incidentally, our K-M survival curve also illustrated that subjects with admission hyperglycemia presented lower survival rate and shorter survival time (log-rank *p* < 0.0001, Figure 3). Cox proportional hazards model was used to further explore the association between BG and all-cause mortality (Table 2). With the use of the low glucose tertile as reference, after adjustment for the confounders of age, sex, and ethnicity (model I), the high glucose tertile was associated with increased risk of 30-day (HR, 95% CI: 3.00, 2.12–4.25), 90-day (HR, 95% CI: 2.65, 1.94–3.61), and in-hospital all-cause mortality (HR, 95% CI: 2.58, 1.76–3.77). Moreover, the middle glucose group was only associated with increased risk for 30-day all-cause mortality (HR, 95% CI: 1.55, 1.06–2.27). In model II, after adjustment for heart rate, cardiac arrest, SBP, DBP, SOFA, SAPS II, GCS, vasopressor, anion gap, white blood cell, bicarbonate, dialysis, and mechanical ventilation on the basis of mode I, the high-BG tertile still remained as an independent predictor of 30-day (HR, 95% CI: 1.50, 1.02–2.19) and 90-day all-cause mortality (HR, 95% CI: 1.42, 1.01–2.00) with the low glucose group as reference.

**Table 2:** HRs (95% CIs) for all-cause mortality against admission blood glucose tertile.

**Variable**	**Crude**		**Model I**		**Model II**	
	**HR (95% CIs)**	***P*-value**	**HR (95% CIs)**	***P*-value**	**HR (95% CIs)**	***P*-value**
**30-Day all-cause mortality**						
**Glucose**						
Tertile 1	1 (ref)		1 (ref)		1 (ref)	
Tertile 2	1.66 (1.14, 2.43)	0.009	1.55 (1.06, 2.27)	0.024	1.18 (0.79, 1.75)	0.416
Tertile 3	3.87 (2.74, 5.46)	<0.001	3.00 (2.12, 4.25)	<0.001	1.50 (1.02, 2.19)	0.037
p for trend	<0.001		<0.001		<0.001	
**90-Day all-cause mortality**						
**Glucose**						
Tertile 1	1 (ref)		1 (ref)		1 (ref)	
Tertile 2	1.48 (1.05, 2.08)	0.026	1.37 (0.97, 1.94)	0.0714	1.07 (0.75, 1.53)	0.707
Tertile 3	3.42 (2.51, 4.65)	<0.001	2.65 (1.94, 3.61)	<0.001	1.42 (1.01, 2.00)	0.043
p for trend	<0.001		<0.001		<0.001	
**In-hospital all-cause mortality**						
**Glucose**						
Tertile 1	1 (ref)		1 (ref)		1 (ref)	
Tertile 2	1.43 (0.94, 2.17)	0.096	1.43 (0.94, 2.18)	0.091	1.17 (0.76, 1.81)	0.472
Tertile 3	3.20 (2.19, 4.67)	<0.001	2.58 (1.76, 3.77)	<0.001	1.37 (0.91, 2.09)	0.135
p for trend	<0.001		<0.001		<0.001	

*Crude model adjusted for none. Model I adjusted for age, gender, and ethnicity. Model II adjusted for age, gender, ethnicity, heart rate, cardiac arrest, systolic blood pressure, diastolic blood pressure, the sequential organ failure assessment score, the Simplified Acute Physiology Score II, Glasgow Coma Scale, vasopressor, anion gap, white blood cell, bicarbonate, dialysis, and mechanical ventilation*.

### Subgroup Analyses

Subgroup analysis was further conducted to assess the association between the admission BG and 30-day all-cause mortality based on comorbidities, treatment, and disease severity scores. Our results showed that different comorbidities (liver disease, hypertension, DM, pneumonia, respiratory failure, and congestive heart failure), treatment (vasopressor and ventilation utilization), and disease severity scores (SOFA score, SAPS II, and GCS) had no significant interactions on the correlation of BG and 30-day all-cause mortality (*p* > 0.05 in all groups) (Table 3).

**Table 3:** Subgroup analysis of the correlation between admission blood glucose and 30-day all-cause mortality in critically ill patients with subarachnoid hemorrhage.

	***N***	**Glucose (mg/dl)**			**p for interaction**
		**Tertile 1**	**Tertile 2**	**Tertile 3**	
Liver diseases					0.877
No	1,248	1.0 (ref)	1.17 (0.78, 1.76)	1.50 (1.01, 2.22)	
Yes	50	1.0 (ref)	0.99 (0.05, 19.27)	1.44 (0.04, 58.43)	
Hypertension					0.337
No	1,246	1.0 (ref)	1.18 (0.78, 1.78)	1.41 (0.95, 2.11)	
Yes	52	1.0 (ref)	0.03 (0.00, 19.74)	15.07 (0.28, 810.23)	
Diabetes					0.489
No	1,131	1.0 (ref)	1.15 (0.76, 1.73)	1.47 (0.98, 2.21)	
Yes	167	1.0 (ref)	1.82 (0.24, 13.86)	2.42 (0.43, 13.54)	
Pneumonia					0.990
No	1,098	1.0 (ref)	1.13 (0.721.76)	1.32 (0.85, 2.04)	
Yes	200	1.0 (ref)	1.11 (0.40, 3.09)	1.40 (0.55, 3.57)	
Respiratory failure					0.934
No	1,116	1.0 (ref)	1.08 (0.69, 1.70)	1.33 (0.86, 2.05)	
Yes	182	1.0 (ref)	1.17 (0.50, 2.71)	1.59 (0.68, 3.69)	
Congestive heart failure					0.351
No	1,197	1.0 (ref)	1.24 (0.81, 1.89)	1.37 (0.90, 2.08)	
Yes	101	1.0 (ref)	0.16 (0.03, 0.79)	0.68 (0.18, 2.61)	
Vasopressor					0.293
No	1,119	1.0 (ref)	1.15 (0.73, 1.82)	1.45 (0.94, 2.26)	
Yes	179	1.0 (ref)	1.10 (0.48, 2.54)	1.48 (0.61, 3.59)	
Ventilation					0.429
No	648	1.0 (ref)	0.37 (0.14, 0.96)	1.09 (0.46, 2.57)	
Yes	650	1.0 (ref)	1.35 (0.85, 2.14)	1.59 (1.02, 2.48)	
SOFA					0.669
≥2.5	649	1.0 (ref)	1.14 (0.73, 1.77)	181 (1.19, 2.75)	
<2.5	649	1.0 (ref)	1.37 (0.56, 3.36)	1.32 (0.58, 2.99)	
SAPS II					0.068
≥30	683	1.0 (ref)	1.30 (0.84, 2.01)	1.49 (0.99, 2.27)	
<30	615	1.0 (ref)	0.58 (0.19, 1.78)	2.03 (0.69, 5.94)	
GCS					0.190
≥14	884	1.0 (ref)	1.52 (0.87, 2.64)	1.82 (1.07, 3.10)	
<14	414	1.0 (ref)	0.75 (0.40, 1.40)	1.04 (0.57, 1.89)	

*N, number; SOFA, sequential organ failure assessment; SAPS II, simplified acute physiology score II; GCS, Glasgow Coma Scale*.

## Discussion

Our study demonstrated that subjects with admission hyperglycemia presented lower survival rate and shorter survival time, and admission hyperglycemia was an independent predictor for 30- and 90-day all-cause mortality in critically ill patients with SAH. In addition, SAH patients with admission BG >142.00 mg/dl (7.91 mmol/L) had dramatically increased 30- and 90-day all-cause mortality.

Glycemic variability (GV) is a measure of the amplitude, frequency, and duration of glycemic fluctuations around mean BG; encompasses both diurnal hyperglycemic peaks and hypoglycemic troughs; and has become a reliable marker of glycemic control (27). To date, the two most frequently used indicators of GV are coefficient of variation and standard deviation of BG measurements (28). Recently, Okazaki et al. retrospectively reviewed 122 SAH patients and reported that increased GV was an independent predictor of unfavorable neurological outcomes in SAH (29). Admittedly, GV is more likely to reflect glycemic fluctuations over a long period of ICU treatment when comparing with isolated BG measurement. But it should be noted that the assessment of GV requires an evaluation of a patient's BG profile attained from multiple readings sampled over time (30). Continuous glucose monitoring is the preferred method for measuring GV. However, due to its costliness and relative invasiveness, continuous glucose monitoring is still a challenging technology to use in a large epidemiological setting (31). Thus, as is generally affordable and easily access, using admission glucose to predict the short- and long-term prognoses and guide for treatment has great clinical significance in SAH patients.

Despite the relevance of hyperglycemia and that poor outcomes in SAH patients have been extensively discussed for years, it is worth to point out that the definition of hyperglycemia and the evaluation indicator of poor outcomes varied among different studies (14, 17, 19, 32, 33). For instance, a Cuban group investigated the predictors of mortality in 64 patients with SAH, and they reported that serum glucose (>7.0 mmol/L) was an independent risk factor of death for those patients by using multivariate logistic regression analysis model (34). McGirt et al. retrieved a prospectively recorded database with 97 SAH patients and demonstrated that persistent perioperative hyperglycemia was an independent predictor of poor outcome evidenced by poor Glasgow Outcome Scale scores (13). Due to the low incidence of SAH with 6–20/100,000 patients per year, the sample size of the previous study was relatively small. Based on the robust sample size, this study is the largest compilation demonstrating the relationship between admission hyperglycemia and 30- and 90-day mortality in SAH patients and also suggests the U-shape between BG and 30- and 90-day mortality. Besides, SAH patients with BG >142.00 mg/dl (7.91 mmol/L) is associated with increased 30- and 90-day all-cause mortality, which provides some internal validity strength to the present study.

The following factors could account for the association between admission hyperglycemia and poor outcome after SAH. Firstly, admission hyperglycemia is closely related to DCI. As the most important complication after SAH, DCI occurs between 3 and 14 days after hemorrhage and can progress to irreversible cerebral infarction, which is the most important cause of morbidity in patients surviving the initial hemorrhage (6, 35). Patients with hyperglycemia on admission have a higher risk of developing DCI and cerebral infarction than patients with normal BG levels (36–38). Secondly, admission hyperglycemia represents the abnormalities of glucose metabolism in DM or preexistent but previously undiagnosed DM. It has been showed that SAH patients with preexistent DM have increased risk of DCI than have SAH patients without preexistent DM, which suggests a link between abnormalities of glucose metabolism and DCI in SAH patients (39, 40). Consistent with previous study, our results also showed that SAH patients with hyperglycemia had higher percentage of comorbidities of DM. Thirdly, subjects with admission hyperglycemia in this study were older, were overweight, and had higher SOFA and SAPS II scores, all of which could be potential confounding factors causing increased 30- and 90-day all-cause mortality in SAH patients in the high tertile. At last, hyperglycemia on admission is reported to be associated with various in-hospital complications including nosocomial infections, impaired wound healing, and respiratory failure, all of which are contributors to poor outcome, while intensive insulin treatment lowers these in-hospital complications (37, 41).

Contrary to hyperglycemia, hypoglycemia was also associated with poor outcomes in SAH patients (42). A retrospective study analyzed 122 SAH patients, finding that over 50% of patients with minimum glucose <8 mmol/L (90 mg/dl) presented unfavorable outcomes at discharge (29). However, our study showed the relationship between hyperglycemia and 30/90-day mortality, and a BG >142.00 mg/dl (7.91 mmol/L) was identified as a threshold for BG levels predicting unfavorable outcome. To date, the optimal glycemic control in SAH patients is still controversial. A retrospective observational study showed that a strictly controlled glucose at 90–120 mg/dl (5.0–6.7 mmol/L) failed to reduce mortality and was associated with an increased incidence of hypoglycemia in SAH (43). Another randomized controlled trial performed in 78 SAH patients after surgical clipping found that intensive insulin therapy (4.4–6.7 mmol/L; 80–120 mg/dl) significantly reduced infection rates as compared with maintaining BG <11.1 mmol/L (200 mg/dl). But the benefit of strict glycemic control on mortality rates was not affected by intensive insulin therapy (38). Based on those findings, a moderate regimen, maintaining glucose levels up to 7.91 mmol/l, might be reasonable in the acute phase after SAH.

Our study has some limitations to consider. Firstly, MIMIC-III database is a large, single-center database that included critically ill patients admitted to ICU. Thus, caution is warranted in extrapolating the positive findings to all patients with SAH. Secondly, we could not exclude the possibility that other unmeasured confounders that could not all be assessed in this study might have affected our findings, such as the incidence of DCI, vasospasm, re-bleeding, and the use of other hemodynamic monitoring techniques in each group. Thirdly, although the association between hyperglycemia and 30- and 90-day all-cause mortality has been observed, this does not prove causality of that relation. Experimental as well as clinical observations are needed to further explore the mechanisms through which hyperglycemia may affect clinical outcome after SAH. Lastly, we only used the admission BG level to predict all-cause mortality. It may be more valuable for prognosis prediction if continuous glucose monitoring would be available in a large epidemiological setting in the future.

## Conclusions

To conclude, admission hyperglycemia is associated with increased risk of adjusted 30- and 90-day all-cause mortality in critically ill patients with SAH and is expected to become a simple and effective marker for prognostic evaluation in these patients.

## Data Availability Statement

The datasets presented in this study can be found in online repositories. The names of the repository/repositories and accession number(s) can be found at: https://mimic.physionet.org.

## Ethics Statement

The studies involving human participants were reviewed and approved by Beth Israel Deaconess Medical Center. To protect patient privacy, all data were de-identified; thus, informed consent was waived by the ethical committee of the Beth Israel Deaconess Medical Center.

## Author Contributions

QZ designed the research, contributed valuable advice, and edited the manuscript. DL and YT conducted the research and collected data. DL wrote the draft of the main manuscript. YT analyzed the data and performed statistical analysis. All authors drafted the manuscript, performed the revision, and approved the final version of the manuscript.

## Conflict of Interest

The authors declare that the research was conducted in the absence of any commercial or financial relationships that could be construed as a potential conflict of interest.

## Publisher's Note

All claims expressed in this article are solely those of the authors and do not necessarily represent those of their affiliated organizations, or those of the publisher, the editors and the reviewers. Any product that may be evaluated in this article, or claim that may be made by its manufacturer, is not guaranteed or endorsed by the publisher.

## Abbreviations

BG: blood glucose
CIs: confidence intervals
DBP: diastolic blood pressure
DM: diabetes mellitus
GCS: Glasgow Coma Scale
GV: glycemic variability
HRs: hazard ratios
ICU: intensive care units
MIMIC III: Medical Information Mart for Intensive Care
SAH: subarachnoid hemorrhage
SAPS II: Simplified Acute Physiology Score II
SBP: systolic blood pressure
SOFA: sequential organ failure assessment
SpO_2_: percutaneous oxygen saturation.
